# EEG distinguishes heroic narratives in ISIS online video propaganda

**DOI:** 10.1038/s41598-020-76711-0

**Published:** 2020-11-11

**Authors:** Keith J. Yoder, Keven Ruby, Robert Pape, Jean Decety

**Affiliations:** 1 Department of Psychology, University of Chicago, Chicago, USA; 2 Department of Political Science, University of Chicago, Chicago, USA; 3 Department of Psychology and Department of Psychiatry and Behavioral Neuroscience, University of Chicago, 5848 South University Avenue, Chicago, IL 60637 USA

**Keywords:** Human behaviour, Neuroscience, Psychology

## Abstract

The Islamic State (ISIS) was uniquely effective among extremist groups in the Middle East at recruiting Westerners. A major way ISIS accomplished this was by adopting Hollywood-style narrative structures for their propaganda videos. In particular, ISIS utilized a heroic martyr narrative, which focuses on an individual’s personal glory and empowerment, in addition to traditional social martyr narratives, which emphasize duty to kindred and religion. The current work presented adult participants (n = 238) video clips from ISIS propaganda which utilized either heroic or social martyr narratives and collected behavioral measures of appeal, narrative transportation, and psychological dispositions (egoism and empathy) associated with attraction to terrorism. Narrative transportation and the interaction between egoism and empathy predicted video recruitment appeal. A subset of adults (n = 80) underwent electroencephalographic (EEG) measurements while watching a subset of the video-clips. Complementary univariate and multivariate techniques characterized spectral power density differences when perceiving the different types of narratives. Heroic videos show increased beta power over frontal sites, and globally increased alpha. In contrast, social narratives showed greater frontal theta, an index of negative feedback and emotion regulation. The results provide strong evidence that ISIS heroic narratives are specifically processed, and appeal to psychological predispositions distinctly from other recruitment narratives.

## Introduction

Online militant propaganda videos by ISIS and other groups are strongly implicated in recruitment to terrorism in the West^1–4^. Indeed, over 80% of ISIS offenders arrested in the US had watched ISIS propaganda^5^. Beyond mere use of social media and high production values, ISIS has pioneered a heroic martyr narrative. Distinct from traditional militant appeals based on duty to and religious obligation to kindred communities under threat (the social martyr narrative), the heroic narrative emphasizes individual benefits, notably personal glory and empowerment through sacrifice and righteous violence. Indeed, evidence indicates ISIS is deliberately using both heroic and social narratives to expand its appeal to different recruitment pools characterized by different psychological dispositions and needs associated with attraction to extremism. While a body of literature links terrorism to dispositional altruism^6–8^, the heroic narrative appears tailor-made to attract egoists. The heroic narrative helps to explain the surge of converts and criminals among western recruits for ISIS^9,10^, as well as the documented lack of religious indoctrination and training prior to mobilization^11^.

Narratives as an explanation for behavior have been extensively studied in psychology^12^, political science^13^, communication science^14^, and criminology^15^, and more recently, the study of violent extremism^16,17^. These studies show that simply having a story can enhance persuasion, and that narrative transportation—becoming engrossed in a story and identifying with characters—acts as a key mechanism. But narratives are distinct from narrativity, and no study has yet to assess how narrative types (like the social and heroic martyr narratives used by ISIS) interact with narrative transportation in shaping subjective perceptions of a propaganda video’s recruitment appeal. Further, most existing studies rely on self-reported persuasion (e.g., from surveys, interviews). Self-reports are an unreliable predictor of persuasion and future behavior under normal circumstances^18^, magnified in the case of sensitive topics like support for extremist ideas and behavior, where subjects have strong incentives to misrepresent their attitudes.

Social neuroscience offers tremendous opportunities to improve our understanding of the impact of specific militant narratives on attitudes and behavior^19^. Some preliminary work on narratives has begun to examine how different types of messages presented in video format are associated with changes in electrical oscillations at the scalp level^20^. Moreover, emerging work in “neuro-forecasting” indicates that examining neural responses in a group of individuals can predict aggregate patterns of group behavior above and beyond subjective ratings^21^. Neural responses related to valuation appear to be especially important for predicting aggregate level responses. For instance, studies using functional magnetic resonance imaging (fMRI) demonstrate that hemodynamic response in ventral striatum (VS) and medial prefrontal cortex (MPFC) predict which advertisements will produce more market share^22^, or which news articles will go viral^23^.

Moreover, recent electroencephalography (EEG) studies have shown that scalp voltage oscillations within the beta and gamma frequency bands, while watching movie trailers, can predict global box office performance^24^. Studies combining EEG and fMRI indicate that these oscillations originate from cortical and subcortical regions which support reward processing, especially VS and MPFC^25,26^. While beta and gamma bands provide some insights into the functioning of appetitive systems, other frequency bands elucidate alternative processes. For instance, increased theta oscillations (4–7 Hz) appear to index a separate neural system important for responding to negative feedback^25^. Theta oscillations, with ostensible generators in anterior cingulate and inferior frontal gyrus, have also been linked to emotion regulation and expectancy violations^27,28^. Finally, oscillations within the alpha band (8–13 Hz) have received much attention as a marker of cortical excitation^29^. Thus, widespread increase in alpha power is thought to index decrease in cortical excitability and activation, while reduction in alpha power reflects increased cortical activation, often related to increased attentional engagement^30–32^.

However, little work has been done to apply insights from EEG research to militant propaganda. Studies of radicalized Muslims overseas find that threats to sacred values and social exclusion trigger similar neural pathways associated with willingness to sacrifice^33–35^—a finding consistent with the operation of the social martyr narrative. Importantly, we are still in the dark about the impact of narrative appeals in militant propaganda in general and particularly whether heroic and social narratives operate along similar or distinct neurological and psychological processes. Understanding the impact of specific narratives is important in future identification of vulnerable populations and possible intervention strategies.

The current study integrates behavioral evaluations, dispositional assessments, and neuroscience measures (EEG) to examine the psychological and neural processes impacted by propaganda videos. This integrative social neuroscience approach advances understanding of processes and mechanisms linking narratives and persuasion at higher resolution than behavioral studies alone.

Results indicate that the heroic and social narratives appeal to individuals with different psychological predispositions. Individuals high on egoism and low on empathy were more likely to rate heroic clips as good for recruiting, as were individuals low on egoism and high on empathy. Recruitment potential was predicted by self-reported narrative transportation, especially among religious men. At the neural level, changes in EEG spectral power measured while participants watch short clips from militant propaganda videos can reliably distinguish between heroic and social martyr narratives. Complementary univariate and multivariate analyses provide converging evidence that messages employing a heroic martyr narrative appear to elicit more appetitive responses and require less attention, while social narratives are more engaging and more likely to elicit changes in spectral power associated with emotion regulation. Taken together, the results suggest that ISIS’s use of both narratives could explain the group’s recruitment success but also potential avenues for improving strategies to countering recruitment efforts by extremist groups.

## Results

### Recruitment appeal and narrative transportation

**Figure 1:**
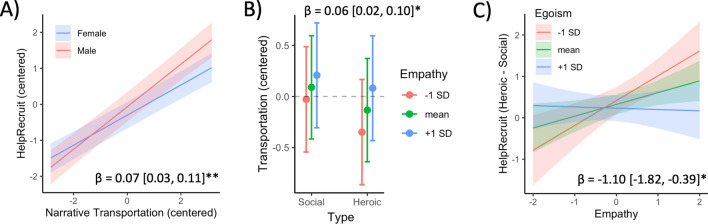
Narrative transportation and empathy. (**A**) Estimated marginal means for the interaction between narrative transportation and sex on HelpRecruit ratings. (**B**) Estimated marginal means for interaction between narrative type and dispositional empathy on individual narrative transportation ratings. (**C**) Estimated marginal means of interaction between dispositional egoism and empathy on preference of heroic narratives (difference between average HelpRecruit ratings of heroic compared to social clips). Standardized parameter estimates and 95% confidence intervals are shown. *FDRp < 0.05; **FDRp < 0.01.

Multilevel linear regressions did not indicate a significant difference in ratings of recruitment appeal in the pool of participants as a whole between heroic and social narrative videos, after correcting for multiple comparisons (Table S2; β = 0.17, 95% CI [0.07, 1.07], uncorrected p = 0.044, FDRp = 0.126) and controlling for age, income, and education. The difference was greater in women (β = 0.07, 95% CI [0.03, 0.11], FDRp = 0.012). Recruitment appeal was positively associated with narrative transportation ratings (β = 0.32, 95% CI [0.28, 0.36], FDRp < 0.001). The effect of narrative transportation was more pronounced in men than women (Fig. 1A; β = 0.07, 95% CI [0.03, 0.11], FDRp = 0.003). Similarly, individuals who reported greater narrative transportation on average were more likely to rate heroic narrative videos as having higher recruitment appeal than social narrative videos (Table S3; β = 0.71, 95% CI [0.33, 1.09], FDRp < 0.001).

### Empathy and egoism

Dispositional empathy and egoism were not independently related to individual recruitment appeal decisions (all p > 0.4). There was weak evidence for a positive association between dispositional empathy and greater average recruitment potential for heroic narratives compared to social narratives (Table S3; β = 0.50, 95% CI [0.07, 0.92], uncorrected p = 0.022, FDRp = 0.080). Interestingly, this effect was qualified by an empathy × egoism interaction (β = − 1.10, 95% CI [− 1.82, − 0.39], FDRp = 0.019; Fig. 1C). Heroic videos were preferred by individuals with high egoism and low empathy, or low egoism and high empathy, while Social videos were preferred by those who are low on both dispositions.

### Empathy and narrative transportation

Individual narrative transportation ratings were higher for individuals with higher dispositional empathy, though this effect became marginally significant after multiple comparison correction (Table S4; β = 0.10, 95% CI [0.02, 0.17], uncorrected p = 0.012, FDRp = 0.052). Empathy dispositions showed a stronger association with narrative transportation when viewing heroic narrative videos (β = 0.06, 95% CI [0.02, 0.10], FDRp = 0.028; Fig. 1B). Individuals with higher dispositional empathy reported equivalent narrative transportation, regardless of clip type, but individuals with lower empathy reported less transportation for heroic videos.

### Impact of different narratives on spectral power

**Figure 2:**
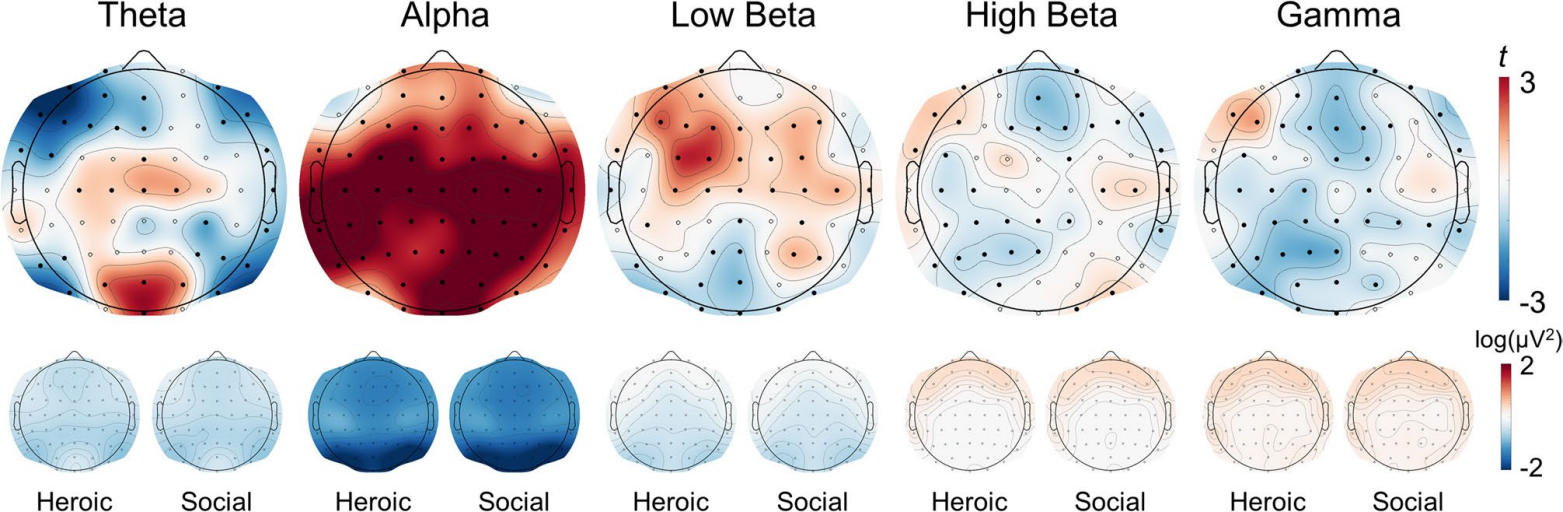
EEG oscillations that differ between heroic and social narratives. Scalp plots of paired t-test comparing log spectral power density (heroic – social). Electrodes with significant differences showed as filled circles (TFCE FWE-p < 0.001). Scalp plots for log power density compared to eyes-closed resting for each condition shown below.

Electrodes with significantly different log power between heroic and social videos were identified in each frequency band (Fig. 2). Within the theta band, heroic videos were associated with reduced power at posterolateral and frontal electrodes but increased power at centro- and posterior medial electrodes. All channels demonstrated greater alpha power for heroic clips, except for the most fronto-lateral electrodes (F7/8, and AF7/8). Heroic videos elicited greater power over frontocentral sites in the low beta band, and greater power over left frontolateral sites across the low beta, high beta, and low gamma bands. In contrast, social videos were associated with great posterior power across all three higher bands, with the broadest effects within the highest frequency band.

### Narrative transportation and spectral power

Linear regressions identified several frequency bands where the differences between heroic and social narratives were associated with participants’ average narrative transportation value. After controlling for clip type, age, and sex, individuals who were more easily transported demonstrated greater global power within the low beta band (β = 0.36, 95% CI [0.16, 0.57], FDRp = 0.007). Similar, smaller effects were also observed for the gamma band (β = 0.31, 95% CI [0.10, 0.52], FDRp = 0.026) and the high beta band, which became marginally significant after multiple comparison correction (β = 0.27, 95% CI [0.07, 0.48], uncorrected p = 0.012, FDRp = 0.052).

### Patterns of spectral density predict narrative type

**Figure 3:**
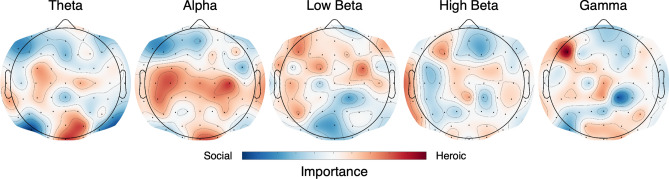
EEG oscillations important for classifying heroic or social narratives. Scalp plots of weights from the linear support vector machine indicating importance of each electrode and frequency band for classifying a video as heroic (red) or social (blue). Values are scaled to the overall maximum beta weight.

The average cross-validated accuracy across participants was 0.636, compared to a chance level of 0.5 (95% bootstrap CI [0.614, 0.658], p < 0.0001). In order to visualize which electrodes and frequency bands were most important for discriminating between clip types, the average beta weights for each subject were averaged, then divided by the overall absolute maximum to scale from − 1 to 1. Scaled importance values were then projected to the scalp (Fig. 3) and show similar distributions to the univariate maps, though the alpha band is much less prominent.

### Religiosity, gender, and decoding accuracy

Participants’ classifier accuracies were regressed on religiosity and narrative transportation separately, controlling for demographics in each case. These multiple regressions revealed an interaction between sex and religiosity that became marginally significant after correction for multiple comparisons (β = 0.55 95% CI [0.06, 1.05], uncorrected *p* = 0.030, FDRp = 0.086). Religiosity was positively related to decoding accuracy in males, but not females. Narrative transportation was not related to decoding accuracy (all p > 0.5).

## Discussion

In the past decade, Islamist terrorist groups have been successful in recruiting members from Europe and North America using propaganda videos posted on the Internet. Why such material is effective remains a complex question, for which social sciences and neuroscience can contribute in elucidating the mechanisms at play. Results from the current behavioral and EEG studies show that the two main narratives that ISIS uses in Western recruitment videos—the heroic and social martyr narratives—are associated with distinct patterns of spectral power and appeal to different combinations of psychological predispositions. Prior studies have shown that simply having a narrative story increases the persuasive appeal of extremist messages^17^, casting doubt on the impact of psychological predispositions on attraction to terrorism^36,37^, and left unanswered the puzzle of the thin role of religiosity among ISIS international recruits^38^.

First, findings are consistent with our theoretical framework, which argues that narrative transportation is a key mechanism underlying the recruitment appeal of ISIS propaganda videos. Behaviorally, subjective narrative transportation ratings were strongly positively related to greater preference for particular propaganda videos. Moreover, narrative transportation was also positively associated with global power in the beta and gamma frequency bands.

Second, heroic and social narrative videos elicited distinct patterns of brain activation in affecting appeal, consistent with these being distinct and distinguishable narrative types. The frontal theta and low beta results provide insights into the affective mechanisms that distinguish between social and heroic narratives. Heroic videos elicited widespread increases in frontal beta, which is consistent with heroic videos being more personally relevant. In contrast, social martyr narratives were associated with increased power at frontolateral sites within the theta band and less global alpha, suggesting the social narratives required greater attention and potential emotion regulation. In previous event-related experiments, brief increases in theta power follow errors and negative feedback, while beta oscillations track positive feedback (e.g.^39,40^). Greater beta power over frontal sites for heroic narratives is also consistent with greater positive expectations. Past work has also demonstrated widespread decreases in alpha power associated with autobiographical memory (e.g.^41^). Thus, heroic videos may be more likely to lead individuals to reflect on their own past experiences.

Third, narrative type may also contribute to enhanced predictions of persuasion and behavior. Previous work has identified frontal beta power as an important factor for neuroforecasting because it likely reflects signal from ventromedial prefrontal cortex, a core value-processing region^21,42^. For example, previous work found that individual beta power elicited by movie trailers predicted global box office performance^24^. Thus, ISIS’s social and heroic propaganda narratives may work by leveraging both of the twin pathways of the Elaboration Likelihood Model of persuasion^43^. Social narratives call individuals to reflect and deliberate on specific details, heroic narratives utilize narratives forms that are both familiar to Western audiences, but also requires fewer attentional resources and offer more promise of personal glory. In the current study, this manifests as a less cortical engagement (greater global alpha power) and more positive expectations (greater frontal beta power).

Fourth, dispositional empathy and egoism, in conjunction with narrative type, resulted in diverse patterns of subjective assessments of militant group appeal, suggesting that the use of social and heroic narratives expands the avenues through which ISIS and other militant groups can enhance the persuasiveness of their propaganda. Individuals with higher dispositional empathy reported greater narrative transportation and a preference for heroic narratives. The positive association between dispositional empathy and preference for heroic martyr narratives may seem contradictory, given that the heroic narrative promises the viewer glory and power, psychic goods not usually associated with empathy. However, empathy is not necessarily associated with general prosociality^44^, since it involves emotion arousal based on others emotions as well as urges to care for the welfare of others^45^, but it is influenced by group dynamics. It is significant that in our study dispositional empathy was associated with a greater tendency to engage with the narrative of the videos and led to higher average HelpRecruit ratings for heroic compared to social videos, which could suggest that the heroic narrative triggered empathy in the sense of emotion contagion where one person’s emotions are aroused by the emotions of another. Future work using disaggregated measures of other-regarding dispositions may disentangle these effects.

At the same time, the current results shed light on part of the reason why counter-narrative strategies have shown no evident success against ISIS. Though social and heroic narratives are associated with reliably distinct neural response patterns, individuals vary in the extent to which they generally prefer one narrative type over the other. By utilizing both social and heroic narratives, ISIS was able to appeal to a broader group of individuals. However, counter-narratives thus far have primary used a one-size-fits all approach under a social narrative logic to emphasize ISIS’s use of extreme violence and harm in order to undermine the legitimacy and support for groups like ISIS^46^. However, the results presented here suggest that for individuals searching for glory and empowerment, these efforts could prove ineffective or even have the opposite effect, reinforcing the opportunities of glory and power at the core of the heroic narrative. Engaging both motivations will be necessary to develop effective counter-messaging.

Finally, religiosity (as practice independent of religion) enhanced narrative transportation and the distinctiveness of heroic and social narratives in the EEG results (as measured by decoding accuracy)—but only for men. Religious male participants demonstrated sharper representational boundaries between heroic and social narratives, which may explain why ISIS uses religious language and images to some extent in all of its recruitment videos, whether social and heroic narrative videos.

The current study provides an important preliminary investigation into the neuropsychological systems which are sensitive to differences between heroic and social martyr narratives. Future work should extend these findings to other populations. Though the clips used in this study did not significantly differ in terms of overall luminance (see “Methods”), it will be beneficial for future work to thoroughly investigate low-level stimulus characteristics that might potentially confound neural findings.

## Conclusion

Overall, these findings advance the general research program to identify how and why militant online video propaganda operates in order to discover improved responses to divert, counteract, and mitigate its effects. The unusual success of ISIS at recruiting from the West appears to rely on their adoption of a unique messaging style, effectively borrowing a page from Hollywood screenwriting to exploit Western-style “heroic” narratives. Framing decisions to join ISIS within a heroic narrative targets different psychological processes which are associated with distinct patterns of neural responses. Whereas social narratives ask individuals to reflect and deliberate on specific details, heroic narratives utilize narratives forms that are both familiar to Western audiences, but also require fewer attentional resources and offer more promise of personal glory. Heroic narratives rely on appeals to positive affect without requiring additional mental effort. Narrative transportation and dispositional empathy thus represent two potential mechanism for the success of heroic narratives at recruiting subsets of Westerners who might otherwise have been missed.

## Methods

### Participants

In total, 231 adult volunteers participated in the video evaluation task. Participants were recruited using physical flyers and digital ads in the Chicagoland area. Of those, 208 provided complete demographic and behavioral data (148 females, M_age_ = 22.6, SD = 6.4, Median = 22.1, Range = 18–56). Participants were invited to return at a later date for the EEG portion of the study until 80 individuals had responded. Exclusion criteria for EEG included history of head injury, active smoking, or active use of psychoactive medication. Four were removed for data with insufficient numbers of clean trials, leaving a final EEG sample of 76 (48 female, M_age_ = 23.7, SD = 6.8). Participants were compensated $20 for the behavior ratings and an additional $50 for the EEG portion. Participants provided informed written consent. All materials and procedures were approved by the Institutional Review Board at the University of Chicago, and the study was conducted in accordance with the ethical guidelines of the Helsinki declaration.

Prior to providing video evaluations, participants completed several online measures including demographic information, age, income, and education. They also completed self-report measures to assess religiosity as well as other-focused and self-focused dispositions. Religiosity was assessed using three items from the Duke University Religiosity Index^47^ which ask participants to indicate the extent to which religious beliefs and spirituality play an important role in their everyday lives. Participants were given the option to report their religious affiliation, if any (see Table S1). There were a wide variety of religions represented in this community sample, so it was not possible to directly compare religions.

Other-regarding dispositions included sub-scales from the Interpersonal Reactivity Index^48^ and the Justice Sensitivity Inventory^49^. The IRI includes subscales for empathic concern, the motivation to appropriately respond to the needs of others, as well as perspective taking, the tendency to adopt another person’s view. The JSI assess an individual’s sensitivity to injustice when they are an observer to, beneficiary of, or perpetrator of the injustice. Each perspective is assessed using 10 statements and participants use a six-point scale to indicate the extent to which they agree or disagree with each statement. Empathic concern, perspective-taking, and the three other-oriented JSI measures were combined into a composite Empathy score.

Dispositional focus on the self was assessed using the Narcissistic Personality Inventory^50^. The NPI is a 40-item self-report measure that assesses seven aspects of narcissism: authority, self-sufficiency, superiority, exhibitionism, exploitativeness, vanity, and entitlement. Participants indicate their agreement or disagreement with each statement.

### Video validations

A collection of 36 (18 each with ostensible heroic or social narrative content) English-language videos produced by ISIS were downloaded from the internet (see Supplementary Tables S5–S7 for details of each clip). Social clips emphasize duty and obligation to defend a threatened community of which the viewer is a member. Heroic clips, in contrast, emphasize opportunities for glory and self-empowerment through action and adventure designed to appeal to viewers with weak or no ties to the threatened community. In a pilot study, 79 adults rated these 36 clips for the degree to which they appealed to duty/obligation (social) or glory/self-empowerment (heroic). Dimension ratings for each narrative were averaged and combined (via subtraction) into an index from which the 7 highest scoring (most heroic) and 7 lowest scoring (most social) clips were selected (duration M = 58.2 s, SD = 14.4, range = 22–78 s).

Clips were cropped to 1280 × 720 resolution with 25 frames per second. RGB values for each frame of each video were converted to YIQ values in accordance with National Television System Committee. A multilevel linear regression modeled frame-wise mean YIQ luminance against narrative type with a random intercept for clip. Heroic and social clips did not significantly differ in terms of mean luminance (β = − 0.26, 95% CI [− 0.58, 0.06], p = 0.296).

Participants viewed the video clips in a random order using the E-Prime presentation software. After viewing each clip, they provided subjective evaluations using a 7-point Likert scale (1 = Disagree, 7 = Agree). Rather than directly ask participants if the video had swayed their opinion about the militant group, participants were asked to respond to a Help Recruit statement: “This video would help the militant group recruit.” Narrative transportation was assessed using three items (the last two reverse-scored): “I could picture myself in the scene of the events described in the video,” “After the video ended, I found it easy to put it out of my mind,” and “I found my mind wandering while watching the video.” Questions were presented in random order.

### Behavioral analysis

Participants subjective evaluations were analyzed using multilevel linear regressions, as implemented in the R package ‘lme4’^51^. First, individual Help Recruit ratings were regressed on narrative transportation ratings and narrative type, controlling for sex, age, education, income, and religiosity. Second, narrative transportation was regressed on dispositional empathy and egoism, again controlling for demographics. A third model was used to characterize which individuals generally believe heroic or social narrative have higher recruitment potential by subtracting average ratings for social clips from heroic clips. Difference scores were then regressed on narrative transportation and the interaction between egoism and empathy, again controlling for demographic information. Age, education, income, and religiosity were mean-centered. Random intercepts were included for participant and for video clip.

### EEG acquisition and analysis

In order to select clips for EEG analysis, an independent sample of 153 adults (79 females) evaluated the extent to which each clip appealed to duty, glory, personal empowerment, or compassion using a 7-point Likert scale (data not presented here). A linear support vector machine (SVM) was trained to classify clips as containing heroic or social narratives based on these ratings. Cross-validated accuracy reached 81% (randomization p < 0.0001) when using all clips. A grid search for all 4-clip (two heroic and two social) subsamples revealed a maximum accuracy of 84% (permutation p < 0.0001). The four videos which produced the highest cross-validated accuracy were taken as exemplars and selected for use in the subsequent EEG study (mean duration = 49 s, range = 34–63 s).

EEG recordings were acquired from 64 sintered Ag/AgCl electrodes embedded in a cap laid out according to the international 10–20 system (EasyCap, Brain Products). No other sensors were attached to the head. Electrode impendences were kept below 25 kΩ in accordance with the manufacturer’s recommendations. Participants completed 5 min of eyes-closed resting in order, then viewed each clip once in randomized order. Data were recorded with reference to Cz and digitized at 2000 Hz. After recording, data were re-referenced to the average of all channels, then bandpass filtered between 0.1 and 50 Hz using an infinite impulse response Butterworth filter. At the same time, a notch filter at 60 Hz was applied to remove line noise. After filtering, blind source separation was performed using infomax independent component analysis (ICA) as implemented in BrainVision Analyzer 2.1 to create 63 independent components. Based on time-course and scalp topography, IC(s) reflecting ocular artifacts (e.g. blink activity, gaze shifts) were identified and removed. Following ocular correction, data were downsampled to 250 Hz and inspected for artifacts. Semi-automated routines identified ostensible artifacts using four criteria: (1) voltage step exceeding 100 µV/ms, (2) voltage change exceeding 200 µV within 200 ms, (3) absolute voltage exceeding 200 µV, and (4) activity of less than 0.5 µV during 100 ms. Sections of data exceeding these criteria were marked, as well as 200 ms preceding and following each artifact.

After artifact detection, each video clip was segmented into non-overlapping 1 s windows. Each segment was tapered using a 10% Hanning window and the power density in each segment was calculated using a fast fourier transform. Power density values for each segment at each channel were then exported within five specific frequency bands^42^: theta (4–8 Hz), alpha (8–12.5 Hz), low beta (15–20 Hz), high beta (25–35 Hz), gamma (35–40 Hz). Participants were required to have at least 20 clean trials per condition (M = 62.3, SD = 10.0).

For univariate analysis, log-transformed power values were averaged at each electrode within each frequency band for each subject for heroic and social videos separately. Paired-samples t-tests were then conducted at each electrode, within each frequency band. Statistical significance was assessed using threshold-free cluster enhancement, a method that takes into account both height (t-statistic) and extent (neighboring electrodes with similar response) of differences in power between heroic and social videos^52,53^. Here, family-wise error was assessed using 5000 Monte Carlo samples, calculated within each frequency band, separately for positive and negative differences.

For classification analysis, log power density within each 1 s window served as samples for training a linear classifier. Thus, each sample contained 320 features (64 channels × 5 frequency bands). Classification analysis was performed using a support vector machine (SVM) with a linear kernel as implemented in fitcecoc in MATLAB 9.2 (R2017a, MathWorks). Prior to training, power density within each frequency band was standardized. When the number of clean segments were not equal between the heroic and social videos, random subsampling was used to equalize trial numbers. Classifier performance was evaluated by the average accuracy over 10 repetitions of tenfold cross-validation. Classifier significance was assess using a randomization test against chance (50%) with 10,000 Monte Carlo simulations. In order to provide an estimate of the stability of the average classifier accuracy, a 95% bootstrap interval was calculated.

## Supplementary information

Supplementary Information.
