# Supplementary material for: EEG distinguishes heroic narratives in ISIS online video propaganda
Source: Sci Rep. 2020 Nov 11;10:19593. doi: 10.1038/s41598-020-76711-0 (PMC7659011; doi:10.1038/s41598-020-76711-0)
Supplement: Supplementary file 1 — Supplementary Information. [file 41598_2020_76711_MOESM1_ESM.pdf]

Supplementary Data for "EEG distinguishes heroic narratives in ISIS online video propaganda"

Keith J. Yoder[1], Keven Ruby[2], Robert Pape[2] and Jean Decety[1*]

1. Department of Psychology, University of Chicago

2. Department of Political Science, University of Chicago

* Dr. Jean Decety – Department of Psychology and Department of Psychiatry and Behavioral Neuroscience, University of Chicago, 5848 South University Avenue, Chicago IL 60637.

E-mail: decety@uchicago.edu

Supplementary Table 1. Reported religious affiliation

Religion	Video Ratings (N = 231)	EEG Study (N = 80)
Christianity	52	11
Islam	5	1
Judaism	12	3
Buddhism	4	1
Hinduism	4	0
Agnosticism	34	9
Atheism	38	10
Other	6	3
Multiple	5	1
No Answer	33	9
(Missing)	38	32

Supplementary Table 2. Model summary for recruitment potential ratings

Fixed Effects	Estimate	β (95% CI)	FDRp	Estimate	β (95% CI)	FDRp
(Intercept)	-0.29		0.2736	-0.3		0.2700
Transportation	0.42	0.32 (0.28 – 0.37)	0.0000	0.42	0.32 (0.28 – 0.36)	0.0000
Male	0.21	0.06 (-0.01 – 0.13)	0.2431	0.23	0.06 (-0.01 – 0.14)	0.1974
Heroic	0.57	0.17 (0.02 – 0.32)	0.1264	0.57	0.17 (0.02 – 0.33)	0.1264
Religiosity	-0.04	-0.03 (-0.09 – 0.04)	0.5856	-0.04	-0.03 (-0.09 – 0.04)	0.5948
Age	-0.01	-0.03 (-0.11 – 0.04)	0.5603	-0.01	-0.04 (-0.11 – 0.04)	0.5603
Education	0.17	0.05 (-0.02 – 0.12)	0.2949	0.18	0.05 (-0.02 – 0.12)	0.2843
Income	-0.09	-0.03 (-0.10 – 0.03)	0.5082	-0.09	-0.03 (-0.10 – 0.03)	0.5196
Transportation * Male	0.17	0.07 (0.03 – 0.11)	0.0029	0.17	0.07 (0.03 – 0.11)	0.0029
Male * Heroic	-0.33	-0.07 (-0.11 – -0.03)	0.0115	-0.33	-0.07 (-0.12 – -0.03)	0.0122
Egoism				-0.03	-0.02 (-0.09 – 0.06)	0.7625
Empathy				0.04	0.02 (-0.06 – 0.09)	0.7443
Heroic * Egoism				0.04	0.01 (-0.03 – 0.06)	0.6249
Heroic * Empathy				0.03	0.01 (-0.03 – 0.05)	0.7278
**Random Effects**	**Variance**			**Variance**		
Residual	1.63			1.63		
Participant	0.50			0.50		
Clip	0.21			0.21		
Marginal R^2	0.148			0.150		
Condition R^2	0.407			0.409		

Estimates from multilevel linear regression. CI: confidence interval. FDRp: False discovery rate corrected p-values.

Supplementary Table 3. Model summary for average recruitment potential difference (Heroic – Social).

Term	Estimates	β (95% CI)	FDRp
(Intercept)	-0.72		0.4091
Transportation	0.89	0.71 (0.33 – 1.09)	0.0030
Male	-0.14	-0.08 (-0.48 – 0.31)	0.6249
Age	0.01	0.16 (-0.27 – 0.59)	0.6249
Education	0.25	0.43 (-0.03 – 0.88)	0.1695
Income	-0.03	-0.08 (-0.54 – 0.38)	0.7829
Empathy	0.29	0.50 (0.07 – 0.92)	0.0798
Egoism	-0.09	-0.19 (-0.59 – 0.22)	0.5603
Transportation * Male	-0.67	-0.75 (-1.51 – 0.01)	0.1492
Empathy * Egoism	-0.32	-1.10 (-1.82 – -0.39)	0.0194
Adjusted R^2	0.271		

Estimates from linear regression. CI: Confidence interval; FDRp: False discovery rate corrected p-value.

Supplementary Table 4. Model summary for narrative transportation ratings

Fixed Effects	Estimate	β (95% CI)	FDRp
(Intercept)	0.09		0.7829
Male	-0.02	-0.01 (-0.08 – 0.06)	0.8393
Heroic	-0.23	-0.09 (-0.37 – 0.19)	0.6748
Egoism	0.02	0.01 (-0.06 – 0.09)	0.7829
Empathy	0.15	0.10 (0.02 – 0.17)	0.0518
Religiosity	0.00	0.00 (-0.07 – 0.07)	0.9660
Age	0.02	0.10 (0.02 – 0.17)	0.0552
Education	-0.17	-0.06 (-0.14 – 0.01)	0.1858
Income	0.02	0.01 (-0.06 – 0.08)	0.7829
Male * Heroic	0.25	0.07 (0.03 – 0.11)	0.0110
Heroic * Egoism	0.06	0.03 (-0.01 – 0.08)	0.1974
Heroic * Empathy	0.13	0.06 (0.02 – 0.10)	0.0278
Random Effects	Variance		
Residual	0.88		
Participant	0.30		
Clip	0.45		
Marginal R^2	0.032		
Condition R^2	0.474		

Estimates from multilevel linear regression. CI: confidence interval. FDRp: False discovery rate corrected p-values.

Supplementary Table 5. Details of clips used in study

Code	Time (s)	Screenshot	Clip Title	Source
H01	49	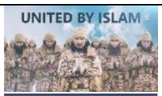	No Respite - The Best of Jihad	No Respite (Al Hayat Media Center, 11/24/2015)
H02	66	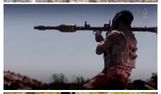	Flames 1 - Best of the Best	Flames of War: Fighting has just begun (Al Hayat Media Center, 09/19/2014)
H03	60	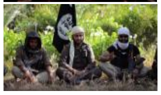	Abu Bara' al-Hindi - Sacrifice Will be Rewarded	There is no life without Jihad (Al Hayat Media Center, 06/19/2014)
H04	78	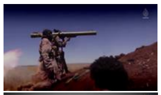	Flames 1 - Tank Hunter [Short]	Flames of War: Fighting has just begun (Al Hayat Media Center, 09/19/2014)
H05	69	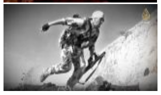	Abu Muslim - Action Sequence [Edit]	The Chosen Few of Different Lands: Abu Muslim from Canada (Al Hayat Media Center, 07/11/2014)
H06*	63	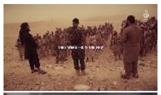	Flames 2 - Few of the Few	Flames of War 2: Until the Final hour (Al Hayat Media Center, 11/29/2017)
H07*	61	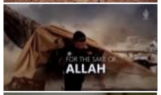	For the Sake of Allah Nashid - Clip 2	Legacy of Prophetic Methodology (Al Hayat Media Center, 08/29/2015)
S01*	34	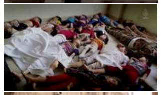	Flames 1 - Atrocity	Flames of War: Fighting has just begun (Al Hayat Media Center, 09/19/2014)
S02	55	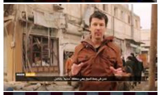	John Cantlie - Airstrikes Aftermath in Halab	From Inside Halab (Al Hayat Media Center, 02/09/2015)
S03	76	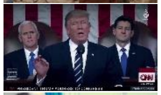	Flames 2 - American Atrocities [Short]	Flames of War 2: Until the Final Hour (Al Hayat Media Center, 11/29/2017)
S04	74	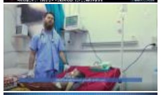	Australian Doctor - Dying Children	The Fertile Nation #2 (Wilayat ar-Raqqah, 07/03/2017)
S05*	38	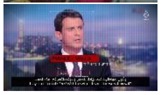	My Vengeance Nashid - French Regime Airstrikes [Short]	My Vengeance (Al Hayat Media Center, 07/05/2016)
S06	54	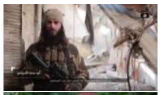	Abu Hamza al-Amriki - Muslim Obligation	We Will Surely Guide Them To Our Ways (Wilayat Ninawa, 05/17/2017)
S07	22	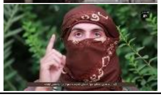	Allah Takes Revenge	And We Made For Their Destruction (Wilayat al-Barakah, 09/22/2017)

H: Heroic; S: Social. * clip used in EEG study

Supplementary Table 6. Clips with ostensibly heroic narratives used in validation

Code	Time (s)	Screenshot	Clip Title	Source
H08	73	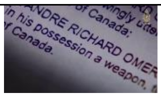	Abu Muslim - Heroic Journey [Short]	The Chosen Few of Different Lands: Abu Muslim from Canada (Al Hayat Media Center, 07/11/2014)
H09	42	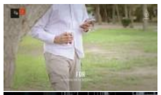	Enemies of Allah Nashid [Short]	An Euch Feinde (Al Hayat Media Center, 04/15/2015)
H10	39	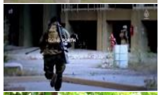	For the Sake of Allah Nashid - Clip 1 [Short]	Legacy of Prophetic Methodology (Al Hayat Media Center, 08/29/2015)
H11	60	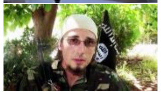	Abu Muslim - Ordinary Life [Short]	The Chosen Few of Different Lands: Abu Muslim from Canada (Al Hayat Media Center, 07/11/2014)
H12	56	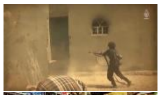	Legacy - Hollywood Battles [Short]	Legacy of Prophetic Methodology (Al Hayat Media Center, 08/29/2015)
H13	69	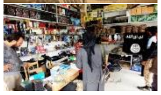	Abu Khaled al-Cambodi - Joining ISIS [Short]	Stories from the Land of the Living: Abu Khaled al-Cambodi from Australia (Al Hayat Media Center, 04/21/2015)
H14	43	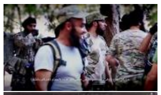	Flames 1 - Few of the Few	Flames of War: Fighting has just begun (Al Hayat Media Center, 09/19/2014)
H15	52	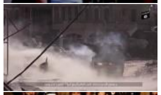	Heroic Testimony Abu Abdullah al-Muhajir	People of Steadfastness (Wilayat Karkuk, 09/06/2017)
H16	60	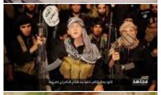	Messages - Al Australi Testimony	Message of the Mujahid #4 (All'tisaam Media Foundation, 10/20/2014)
H17	72	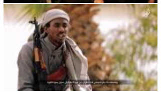	Abu Khaled of Trinadad & Tobago - Transformation in Jihad	Those Who Have Believed and Emigrated (Wilayat ar-Raqqah, 11/05/2015)
H18	57	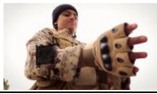	Blood for Blood Nashid - Child Fighters	Blood for Blood (Al Hayat Media Center, 04/29/2016)

Supplementary Table 7. Clips with ostensibly social narratives used in validation

Code	Time (s)	Screenshot	Clip Title	Source
S08	62	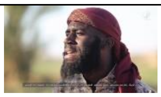	Abu Salih al-Amriki - Revenge for Atrocities [Short]	You Are Not Held Responsible Except For Yourself (Wilayat al-Furat, 06/19/2016)
S09	53	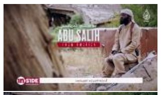	Abu Salih al-Amriki - Answer the Call 1	Inside the Caliphate #6 (Al Hayat Media Center, 12/27/2017)
S10	35	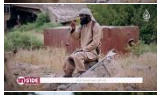	Abu Salih al-Amriki - Answer the Call 2	Inside the Caliphate #6 (Al Hayat Media Center, 12/27/2017)
S11	58	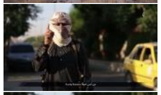	You Must Fight Them - Testimony Clip 2	You Must Fight Them O Muwahid (Wilayat Ar-Raqqah, 11/26/2016)
S12	43	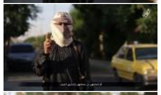	You Must Fight Them - Testimony Clip 1	You Must Fight Them O Muwahid (Wilayat Ar-Raqqah, 11/26/2016)
S13	55	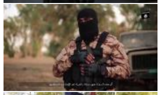	Abu Abdullah al-Muhajir - Testimony	People of Steadfastness (Wilayat Karkuk, 09/06/2017)
S14	60	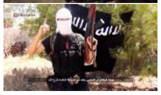	Testimony - Come to Defend Islamic Land	A Window Upon the Land of Epic Battles #8 (All'tisaam Media Foundation, 11/18/2013)
S15	39	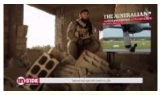	Inside 2 - Australi Call to Aid Brothers	Inside the Caliphate #2 (Al Hayat Media Center, 08/07/2017)
S16	60	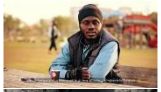	Al Ghani - Defend Islam	From Humiliation to Glory (Wilayat Tarabulus, 05/29/2016)
S17	58	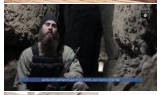	Australian Doctor - Fighter in Cave	The Fertile Nation #2 (Wilayat ar-Raqqah, 07/03/2017)
S18	54	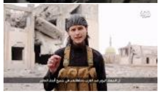	Messages - Al Canadi on Muslim Duty to Retaliate [Short]	Message of The Mujahid 5: Abu Anwar Al Canadi (Al I'tisaam Media, 12/07/2014)
